# (*Z*)-Ethyl 2-hy­droxy-4-oxo-4-(1,4,5,6,8-penta­meth­oxy­naphthalen-2-yl)but-2-enoate

**DOI:** 10.1107/S1600536812049203

**Published:** 2012-12-08

**Authors:** Adushan Pillay, Sanaz Khorasani, Charles B. de Koning

**Affiliations:** a Molecular Sciences Institute, School of Chemistry, University of the Witwatersrand, PO Wits 2050, Johannesburg, South Africa

## Abstract

The title compound, C_21_H_24_O_9_, crystallizes with two independent mol­ecules in the asymmetric unit which are almost centrosymmetrically related to each other. The ethano­ate group in one of the two mol­ecules is disordered over two positions with a site-occupation factor of 0.880 (7) for the major occupied site. In the crystal, the 1,3-diketone group exists in the keto–enol isomeric form due to the stabilizing effect of the intra­molecular O—H⋯O hydrogen bond present in this form. The compound packs as a layered structure in which C—H⋯π and C—H⋯O inter­actions are present within and between the layers.

## Related literature

For the synthesis of the title compound, see: de Koning *et al.* (1991 ▶). This forms part of our research programme directed towards the synthesis of the natural phytotoxic naphtho­quinone, marticin, see: Pillay *et al.* (2012 ▶).
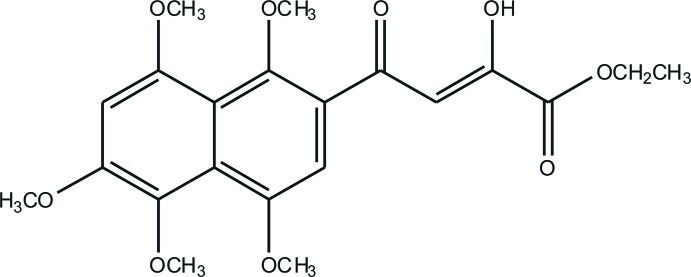


## Experimental

### 

#### Crystal data

C_21_H_24_O_9_
*M* *_r_* = 420.40
Triclinic, 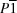

*a* = 6.8019 (4) Å
*b* = 12.3042 (6) Å
*c* = 24.3971 (12) Å
α = 100.002 (4)°
β = 93.080 (4)°
γ = 98.748 (4)°
*V* = 1980.67 (18) Å^3^
*Z* = 4
Mo *K*α radiation
μ = 0.11 mm^−1^
*T* = 173 K
0.39 × 0.16 × 0.07 mm

#### Data collection

Bruker APEXII CCD diffractometer
19594 measured reflections
7793 independent reflections
3172 reflections with *I* > 2σ(*I*)
*R*_int_ = 0.083

#### Refinement

*R*[*F* ^2^ > 2σ(*F* ^2^)] = 0.053
*wR*(*F* ^2^) = 0.118
*S* = 0.84
7793 reflections
572 parameters
54 restraints
H-atom parameters constrained
Δρ_max_ = 0.21 e Å^−3^
Δρ_min_ = −0.25 e Å^−3^

### 

Data collection: *APEX2* (Bruker, 2005 ▶); cell refinement: *SAINT-NT* (Bruker, 2005 ▶); data reduction: *SAINT-NT*; program(s) used to solve structure: *SHELXS97* (Sheldrick, 2008 ▶); program(s) used to refine structure: *SHELXL97* (Sheldrick, 2008 ▶); molecular graphics: *PLATON* (Spek, 2009 ▶) and *SCHAKAL99* (Keller, 1999 ▶); software used to prepare material for publication: *WinGX* (Farrugia, 1999 ▶) and *PLATON*.

## Supplementary Material

Crystal structure: contains datablock(s) global, I. DOI: 10.1107/S1600536812049203/bt6876sup1.cif

Structure factors: contains datablock(s) I. DOI: 10.1107/S1600536812049203/bt6876Isup2.hkl

Supplementary material file. DOI: 10.1107/S1600536812049203/bt6876Isup3.cml

Additional supplementary materials:  crystallographic information; 3D view; checkCIF report

## Figures and Tables

**Table 1:** Hydrogen-bond geometry (Å, °) *Cg*1, *Cg*2 and *Cg*3 are the centroids of the C1*A*/C2*A*/C7*A*–C10*A*, C2*A*–C7*A* and C2*B*–C7*B* rings, respectively.

*D*—H⋯*A*	*D*—H	H⋯*A*	*D*⋯*A*	*D*—H⋯*A*
O7*A*—H7*A*⋯O6*A*	0.84	1.77	2.499 (3)	144
O7*B*—H7*B*⋯O6*B*	0.84	1.77	2.497 (3)	143
C17*B*—H17*D*⋯O6*B* ^i^	0.98	2.43	3.234 (4)	139
C19*A*—H19*B*⋯O9*B* ^ii^	0.98	2.45	3.419 (4)	168
C19*B*—H19*E*⋯O9*A* ^iii^	0.98	2.47	3.440 (5)	172
C20*A*—H20*B*⋯O4*B* ^iv^	0.98	2.50	3.384 (4)	150
C20*B*—H20*E*⋯O4*A* ^v^	0.98	2.57	3.382 (4)	140
C20*B*—H20*F*⋯O9*B* ^i^	0.98	2.58	3.460 (4)	150
C18*A*—H18*A*⋯*Cg*3^vi^	0.98	2.66	3.486 (3)	142
C18*A*—H18*C*⋯*Cg*1^vi^	0.98	2.92	3.844 (3)	158
C18*B*—H18*F*⋯*Cg*2^vii^	0.98	2.66	3.522 (3)	147
C21*A*—H21*A*⋯*Cg*2^vii^	0.98	2.96	3.845 (3)	150

Symmetry codes: (i) 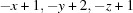; (ii) 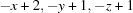; (iii) 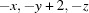; (iv) 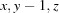; (v) 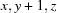; (vi) 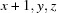; (vii) 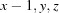.

**Fig. 1.**
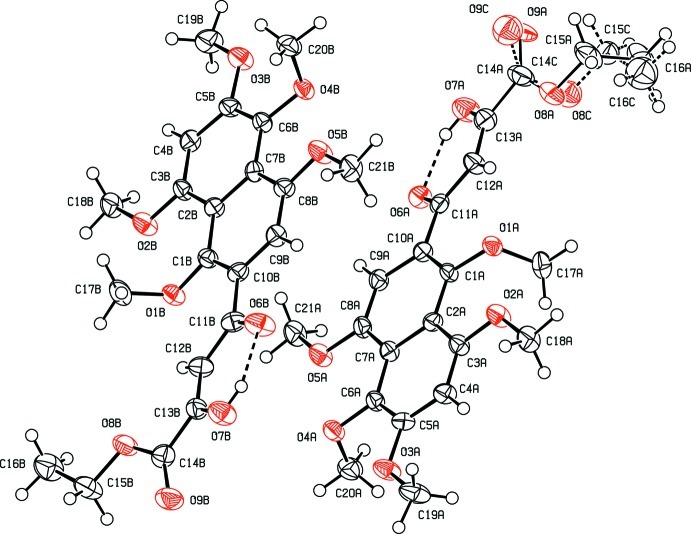
The molecular structure of (I), showing the atomic numbering scheme and intramolecular hydrogen bonding. Displacement ellipsoids are drawn at the 50% probability level.

**Fig. 2.**
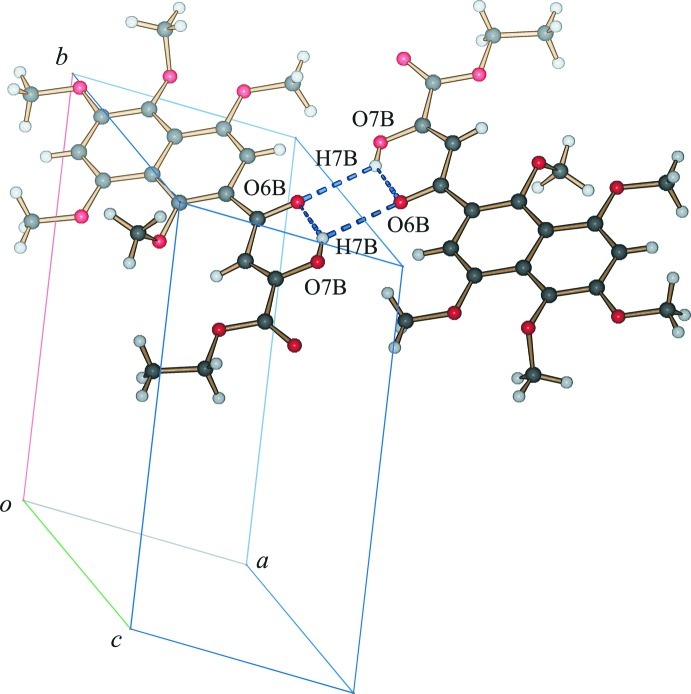
Intermolecular hydrogen bonding between a pair of B molecules related by a centre of inversion. Also shown is the intramolecular hydrogen bonding within each molecule.

**Fig. 3.**
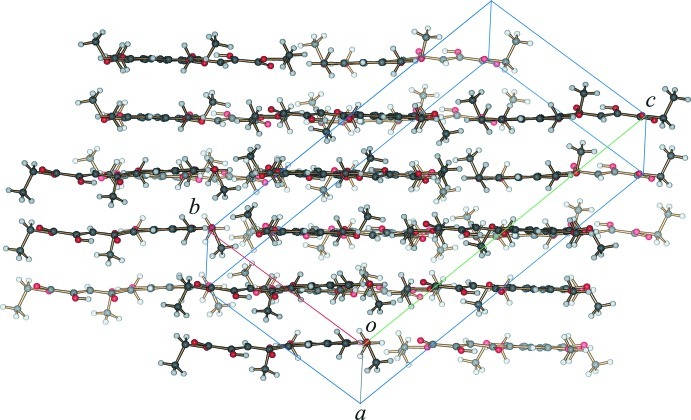
Diagram showing the layered packing of molecules in the structure. C—H···O and C—H···π interactions are present within and between the layers.

**Fig. 4.**
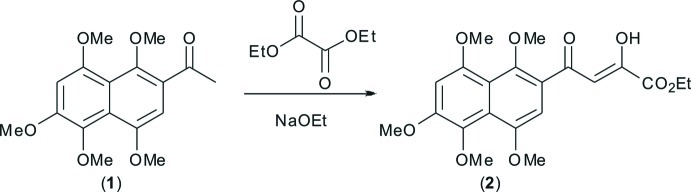
Reaction scheme for the synthesis of the title compound.

## supplementary crystallographic information

### Comment

As part of our research programme directed towards the synthesis of the natural phytotoxic naphthoquinone, marticin (Pillay *et al.*, 2012), we synthesized the title compound in one step from 1-(1,4,5,6,8-pentamethoxynaphthalen-2-yl)ethanone (1) (de Koning *et al.*, 1991). This was achieved as shown in Fig. 4 by treating (1) with diethyl oxalate with sodium ethoxide in THF to afford the title compound (2) as a bright red crystalline solid.

The title organic compound crystallizes in the space group *P* -1 with two independent molecules in the asymmetric unit which are labelled as A and B in Fig. 1. The molecules are almost centrosymmetrically related to each other, with the symmetry being broken by the ethanoate group of molecule A being disordered over two positions. The crystal structure shows that the 1,3-diketone moiety exists as the keto-enol isomeric form, with the enol-ether form being occupied due to the presence of intramolecular hydrogen bonding (Table 1). Strong intermolecular hydrogen bonding is present between pairs of B molecules in which H7B, which is involved in the intramolecular hydrogen bond with O6B, also interacts with O6B of another molecule related by a centre of inversion (Fig. 2.; Table 1). Molecule A is not involved in any strong intermolecular hydrogen bonding. Molecules in the structure pack as a layers (Fig. 3) with C—H···O and C—H···π interactions acting within and between the layers.

### Experimental

To a stirred solution of 1-(1,4,5,6,8-pentamethoxynaphthalen-2-yl)ethanone (0.500 g, 1.62 mmol) and diethyl oxalate (0.474 g, 3.24 mmol, 2.0 equiv.) in dry tetrahydrofruan (50 ml) at 0 *^o^*C, sodium ethoxide (0.220 g, 3.24 mmol, 2.0 equiv.) was slowly added. The reaction mixture was then stirred vigorously for 3 h at RT before being acidified with an aqueous solution of hydrochloric acid (20 ml, 2.0 *M*). Ethyl acetate (20 ml) was then added to the mixture. The organic layer was then separated, dried over anhydrous magnesium sulfate, filtered and concentrated under reduced pressure. Column chromatography (eluant 10% ethyl acetate/hexane) of the residue afforded (*Z*)-ethyl 2-hydroxy-4-oxo-4-(1,4,5,6,8-pentamethoxynaphthalene-2-yl)but-2-enoate as a red solid (0.613 g, 90%); m.p. 117–118 *^o^*C; *R_f_* 0.55 (20% ethyl acetate/hexane); IR (film): *V*_max_ = 3120 cm^-1^ (w, br, OH), 2979 cm^-1^ (w, br, OH), 1818 cm^-1^ (s, C=O), 1594 cm^-1^ (m, C—OH), 1471 cm^-1^ (m, C=C), 1371 cm^-1^ (m, C=C), 1317 cm^-1^ (m, C=C), 1252 cm^-1^ (s, C—O—C), 1222 (s, C—O—C), 1183 cm^-1^ (s, C—O—C),; ^1^H NMR (300 MHz, CDCl_3_) δ_H_ 7.59 (1 H, s, H-2), 7.22 (1 H, s, H-6), 6.79 (1 H, s, COCH=C(OH)CO_2_CH_2_CH_3_), 4.38 (2 H, q, *J* 7.1, COCH=C(OH)CO_2_CH_2_CH_3_), 4.04, 4.02, 3.99, 3.82, 3.78 (each 3 H, s, *OCH_3_*), 1.38 (3 H, t, *J* 3.3, COCH=C(OH)CO_2_CH_2_CH_3_); ^13^C NMR (300 MHz, CDCl_3_) δ_C_ 192.5 (C=O), 166.7 (COCH=C(OH)CO_2_CH_2_CH_3_), 162.7 (COCH=C(OH)CO_2_CH_2_CH_3_), 154.8 (ArC-O), 154.1 (ArC-O), 153.1 (ArC-O), 152.2 (ArC-O), 138.2 (ArC-O), 126.7 (C-1), 123.6 (C-3a), 116.6 (C-7a), 105.0 (C-2), 103.8 (C-6), 97.9 (COCH=C(OH)CO_2_CH_2_CH_3_), 63.8 (OCH_3_), 62.3 (COCH=C(OH)CO_2_CH_2_CH_3_), 61.9, 56.9, 56.8, 56.7 (4 *x* OCH_3_), 14.1 (COCH=C(OH)CO_2_CH_2_CH_3_); HR-TOF-MS: m/*z* found 421.1499, [M—H]^+^ (calculated for C_21_H_25_O_9_, 421.1487).

### Refinement

All H atoms attached to carbon were positioned geometrically, and allowed to ride on their parent atoms, with C—H and O—H bond lengths of 0.95 Å (CH), 0.98 Å (CH_3_) or 0.84 Å (OH), and isotropic displacement parameters set to 1.2 (CH) or 1.5 times (CH_3_ and OH) the *U*_eq_ of the parent atom. The ethanoate group on molecule A was found to be disordered and as a consequence refined over two positions using SIMU, DELU, EXYZ, EADP and SADI constraints. The final occupancies for the two positions were 0.880 (7) and 0.120 (7).

### Crystal data

**Table:**

C_21_H_24_O_9_	*Z* = 4
*M**_r_* = 420.40	*F*(000) = 888
Triclinic, *P*1	*D*_x_ = 1.410 Mg m^−^^3^
Hall symbol: -P 1	Mo *K*α radiation, λ = 0.71073 Å
*a* = 6.8019 (4) Å	Cell parameters from 1943 reflections
*b* = 12.3042 (6) Å	θ = 2.6–25.0°
*c* = 24.3971 (12) Å	µ = 0.11 mm^−^^1^
α = 100.002 (4)°	*T* = 173 K
β = 93.080 (4)°	Needle, red
γ = 98.748 (4)°	0.39 × 0.16 × 0.07 mm
*V* = 1980.67 (18) Å^3^	

### Data collection

**Table:**

Bruker APEXII CCD diffractometer	3172 reflections with *I* > 2σ(*I*)
Radiation source: fine-focus sealed tube	*R*_int_ = 0.083
Graphite monochromator	θ_max_ = 26.0°, θ_min_ = 1.7°
φ and ω scans	*h* = −8→8
19594 measured reflections	*k* = −15→15
7793 independent reflections	*l* = −30→30

### Refinement

**Table:**

Refinement on *F*^2^	Primary atom site location: structure-invariant direct methods
Least-squares matrix: full	Secondary atom site location: difference Fourier map
*R*[*F*^2^ > 2σ(*F*^2^)] = 0.053	Hydrogen site location: inferred from neighbouring sites
*wR*(*F*^2^) = 0.118	H-atom parameters constrained
*S* = 0.84	*w* = 1/[σ^2^(*F*_o_^2^) + (0.0343*P*)^2^] where *P* = (*F*_o_^2^ + 2*F*_c_^2^)/3
7793 reflections	(Δ/σ)_max_ = 0.001
572 parameters	Δρ_max_ = 0.21 e Å^−^^3^
54 restraints	Δρ_min_ = −0.25 e Å^−^^3^

### Special details

**Table:**

Geometry. All e.s.d.'s (except the e.s.d. in the dihedral angle between two l.s. planes) are estimated using the full covariance matrix. The cell e.s.d.'s are taken into account individually in the estimation of e.s.d.'s in distances, angles and torsion angles; correlations between e.s.d.'s in cell parameters are only used when they are defined by crystal symmetry. An approximate (isotropic) treatment of cell e.s.d.'s is used for estimating e.s.d.'s involving l.s. planes.
Refinement. Refinement of *F*^2^ against ALL reflections. The weighted *R*-factor *wR* and goodness of fit *S* are based on *F*^2^, conventional *R*-factors *R* are based on *F*, with *F* set to zero for negative *F*^2^. The threshold expression of *F*^2^ > σ(*F*^2^) is used only for calculating *R*-factors(gt) *etc*. and is not relevant to the choice of reflections for refinement. *R*-factors based on *F*^2^ are statistically about twice as large as those based on *F*, and *R*- factors based on ALL data will be even larger.

### Fractional atomic coordinates and isotropic or equivalent isotropic displacement parameters (Å^2^)

**Table:**

	*x*	*y*	*z*	*U*_iso_*/*U*_eq_	Occ. (<1)
C1A	0.7086 (4)	0.6467 (2)	0.10802 (12)	0.0245 (8)	
C2A	0.7846 (4)	0.6053 (2)	0.15505 (12)	0.0247 (8)	
C3A	0.9780 (5)	0.6484 (2)	0.18382 (13)	0.0274 (8)	
C4A	1.0511 (5)	0.6025 (2)	0.22628 (12)	0.0293 (8)	
H4A	1.1805	0.6317	0.2441	0.035*	
C5A	0.9352 (5)	0.5130 (3)	0.24321 (13)	0.0282 (8)	
C6A	0.7459 (5)	0.4692 (2)	0.21838 (12)	0.0259 (8)	
C7A	0.6673 (4)	0.5137 (2)	0.17411 (12)	0.0244 (8)	
C8A	0.4709 (5)	0.4721 (3)	0.14548 (13)	0.0284 (8)	
C9A	0.4045 (5)	0.5152 (2)	0.10176 (12)	0.0274 (8)	
H9A	0.2747	0.4859	0.0842	0.033*	
C10A	0.5233 (4)	0.6030 (2)	0.08142 (12)	0.0244 (8)	
C11A	0.4404 (5)	0.6341 (3)	0.03013 (13)	0.0269 (8)	
C12A	0.5123 (5)	0.7360 (3)	0.01041 (12)	0.0320 (9)	
H12A	0.6240	0.7863	0.0299	0.038*	
C13A	0.4219 (5)	0.7599 (3)	−0.03533 (14)	0.0363 (9)	
C14A	0.4888 (6)	0.8667 (3)	−0.05716 (15)	0.0437 (9)	0.880 (7)
C15A	0.7626 (7)	1.0144 (3)	−0.0550 (2)	0.0586 (16)	0.880 (7)
H15A	0.8505	1.0631	−0.0238	0.070*	0.880 (7)
H15B	0.6543	1.0549	−0.0648	0.070*	0.880 (7)
C16A	0.8787 (8)	0.9900 (4)	−0.1037 (2)	0.0657 (18)	0.880 (7)
H16A	0.9349	1.0602	−0.1150	0.099*	0.880 (7)
H16B	0.7912	0.9425	−0.1347	0.099*	0.880 (7)
H16C	0.9871	0.9510	−0.0938	0.099*	0.880 (7)
O8A	0.6766 (4)	0.9106 (3)	−0.03759 (16)	0.0469 (10)	0.880 (7)
O9A	0.3818 (5)	0.9035 (3)	−0.08750 (19)	0.0591 (13)	0.880 (7)
C14C	0.4888 (6)	0.8667 (3)	−0.05716 (15)	0.0437 (9)	0.120 (7)
C15C	0.750 (2)	0.971 (2)	−0.0955 (11)	0.035 (7)*	0.120 (7)
H15E	0.6642	1.0293	−0.0880	0.042*	0.120 (7)
H15F	0.7428	0.9443	−0.1363	0.042*	0.120 (7)
C16C	0.958 (4)	1.018 (3)	−0.0738 (15)	0.069 (13)*	0.120 (7)
H16G	1.0093	1.0779	−0.0934	0.103*	0.120 (7)
H16H	1.0407	0.9587	−0.0799	0.103*	0.120 (7)
H16I	0.9627	1.0477	−0.0338	0.103*	0.120 (7)
O8C	0.6804 (13)	0.8774 (15)	−0.0674 (10)	0.046 (5)*	0.120 (7)
O9C	0.371 (3)	0.924 (2)	−0.0690 (15)	0.059 (11)*	0.120 (7)
C17A	0.9644 (5)	0.6896 (3)	0.05027 (13)	0.0385 (9)	
H17A	1.0490	0.6468	0.0688	0.058*	
H17B	1.0480	0.7528	0.0390	0.058*	
H17C	0.8902	0.6411	0.0171	0.058*	
C18A	1.2844 (4)	0.7789 (3)	0.19191 (13)	0.0377 (9)	
H18A	1.2816	0.8059	0.2320	0.057*	
H18B	1.3455	0.8403	0.1744	0.057*	
H18C	1.3626	0.7179	0.1861	0.057*	
C19A	1.1951 (5)	0.5070 (3)	0.31297 (14)	0.0445 (10)	
H19A	1.2947	0.4988	0.2856	0.067*	
H19B	1.2208	0.4648	0.3424	0.067*	
H19C	1.2034	0.5862	0.3295	0.067*	
C20A	0.6807 (5)	0.2757 (3)	0.21377 (14)	0.0409 (9)	
H20A	0.6351	0.2610	0.1740	0.061*	
H20B	0.6098	0.2179	0.2318	0.061*	
H20C	0.8245	0.2748	0.2180	0.061*	
C21A	0.1682 (4)	0.3393 (3)	0.13505 (13)	0.0371 (9)	
H21A	0.0842	0.3976	0.1362	0.056*	
H21B	0.1033	0.2790	0.1526	0.056*	
H21C	0.1871	0.3094	0.0961	0.056*	
O1A	0.8265 (3)	0.73063 (16)	0.08806 (8)	0.0274 (5)	
O2A	1.0851 (3)	0.73912 (17)	0.16741 (8)	0.0352 (6)	
O3A	0.9989 (3)	0.46456 (17)	0.28566 (9)	0.0378 (6)	
O4A	0.6406 (3)	0.38328 (17)	0.23943 (8)	0.0311 (6)	
O5A	0.3595 (3)	0.38650 (17)	0.16480 (8)	0.0360 (6)	
O6A	0.2923 (3)	0.56832 (17)	0.00277 (8)	0.0317 (6)	
O7A	0.2659 (3)	0.6961 (2)	−0.06553 (9)	0.0450 (7)	
H7A	0.2301	0.6399	−0.0510	0.068*	
C1B	0.3209 (5)	0.8636 (2)	0.39125 (12)	0.0268 (8)	
C2B	0.2118 (4)	0.9010 (3)	0.34798 (12)	0.0260 (8)	
C3B	0.0137 (5)	0.8495 (2)	0.32514 (13)	0.0276 (8)	
C4B	−0.0842 (4)	0.8891 (2)	0.28420 (12)	0.0282 (8)	
H4B	−0.2163	0.8545	0.2704	0.034*	
C5B	0.0082 (5)	0.9796 (3)	0.26257 (13)	0.0285 (8)	
C6B	0.2003 (4)	1.0317 (2)	0.28214 (12)	0.0250 (8)	
C7B	0.3025 (4)	0.9964 (2)	0.32591 (12)	0.0246 (8)	
C8B	0.4976 (4)	1.0515 (3)	0.35055 (12)	0.0274 (8)	
C9B	0.5930 (4)	1.0143 (2)	0.39183 (12)	0.0265 (8)	
H9B	0.7211	1.0531	0.4071	0.032*	
C10B	0.5082 (4)	0.9193 (3)	0.41316 (12)	0.0266 (8)	
C11B	0.6317 (5)	0.8932 (3)	0.45984 (13)	0.0318 (8)	
C12B	0.5872 (5)	0.7955 (3)	0.48512 (12)	0.0317 (8)	
H12B	0.4773	0.7389	0.4701	0.038*	
C13B	0.7032 (5)	0.7848 (3)	0.53055 (13)	0.0315 (8)	
C14B	0.6633 (5)	0.6875 (3)	0.55955 (14)	0.0348 (9)	
C15B	0.4164 (5)	0.5460 (3)	0.57949 (14)	0.0468 (10)	
H15C	0.4399	0.5735	0.6203	0.056*	
H15D	0.4960	0.4857	0.5691	0.056*	
C16B	0.1988 (5)	0.5027 (3)	0.56370 (15)	0.0514 (11)	
H16D	0.1224	0.5640	0.5725	0.077*	
H16E	0.1535	0.4439	0.5847	0.077*	
H16F	0.1785	0.4720	0.5236	0.077*	
C17B	0.1232 (5)	0.7820 (3)	0.45533 (13)	0.0405 (9)	
H17D	0.1955	0.8402	0.4853	0.061*	
H17E	0.0933	0.7116	0.4692	0.061*	
H17F	−0.0018	0.8046	0.4432	0.061*	
C18B	−0.2757 (4)	0.7101 (3)	0.32455 (13)	0.0366 (9)	
H18D	−0.3620	0.7670	0.3323	0.055*	
H18E	−0.3215	0.6473	0.3430	0.055*	
H18F	−0.2813	0.6834	0.2842	0.055*	
C19B	−0.2761 (4)	0.9696 (3)	0.19809 (14)	0.0422 (10)	
H19D	−0.2764	0.8902	0.1833	0.063*	
H19E	−0.3183	1.0064	0.1680	0.063*	
H19F	−0.3686	0.9765	0.2275	0.063*	
C20B	0.2339 (5)	1.2213 (2)	0.27309 (13)	0.0403 (9)	
H20D	0.0885	1.2153	0.2680	0.060*	
H20E	0.2968	1.2743	0.2510	0.060*	
H20F	0.2788	1.2479	0.3127	0.060*	
C21B	0.7722 (4)	1.2003 (3)	0.35457 (13)	0.0387 (9)	
H21D	0.7672	1.2272	0.3946	0.058*	
H21E	0.8154	1.2638	0.3363	0.058*	
H21F	0.8670	1.1476	0.3491	0.058*	
O1B	0.2440 (3)	0.76673 (16)	0.40899 (8)	0.0307 (6)	
O2B	−0.0734 (3)	0.75818 (17)	0.34535 (8)	0.0358 (6)	
O3B	−0.0784 (3)	1.02174 (18)	0.22093 (9)	0.0380 (6)	
O4B	0.2889 (3)	1.11377 (17)	0.25499 (8)	0.0293 (5)	
O5B	0.5775 (3)	1.14496 (18)	0.33067 (9)	0.0376 (6)	
O6B	0.7876 (3)	0.96174 (18)	0.47859 (9)	0.0434 (7)	
O7B	0.8649 (3)	0.85584 (19)	0.55340 (9)	0.0433 (6)	
H7B	0.8847	0.9086	0.5356	0.065*	
O8B	0.4734 (3)	0.63665 (18)	0.54955 (9)	0.0403 (6)	
O9B	0.7878 (4)	0.6589 (2)	0.58861 (10)	0.0492 (7)	

### Atomic displacement parameters (Å^2^)

**Table:**

	*U*^11^	*U*^22^	*U*^33^	*U*^12^	*U*^13^	*U*^23^
C1A	0.027 (2)	0.0197 (18)	0.0280 (19)	0.0018 (15)	0.0058 (16)	0.0087 (15)
C2A	0.0191 (18)	0.0271 (19)	0.0273 (19)	0.0021 (15)	−0.0025 (15)	0.0064 (15)
C3A	0.0266 (19)	0.0256 (19)	0.0281 (19)	−0.0021 (15)	−0.0009 (16)	0.0067 (15)
C4A	0.0240 (19)	0.031 (2)	0.0302 (19)	−0.0008 (15)	−0.0084 (16)	0.0073 (16)
C5A	0.031 (2)	0.0256 (19)	0.029 (2)	0.0068 (16)	−0.0031 (17)	0.0074 (16)
C6A	0.0255 (19)	0.0254 (19)	0.0271 (19)	0.0006 (15)	0.0037 (16)	0.0081 (15)
C7A	0.0235 (19)	0.0233 (18)	0.0247 (19)	−0.0003 (15)	0.0004 (16)	0.0035 (15)
C8A	0.026 (2)	0.0273 (19)	0.032 (2)	0.0008 (15)	0.0077 (17)	0.0085 (16)
C9A	0.0216 (19)	0.0292 (19)	0.030 (2)	−0.0001 (15)	0.0008 (16)	0.0064 (16)
C10A	0.0205 (18)	0.0267 (18)	0.0245 (19)	0.0005 (15)	−0.0021 (15)	0.0048 (15)
C11A	0.0246 (19)	0.031 (2)	0.0271 (19)	0.0096 (15)	0.0051 (16)	0.0048 (16)
C12A	0.036 (2)	0.034 (2)	0.027 (2)	0.0022 (16)	0.0009 (17)	0.0113 (16)
C13A	0.038 (2)	0.042 (2)	0.031 (2)	0.0115 (18)	0.0047 (19)	0.0093 (18)
C14A	0.062 (2)	0.041 (2)	0.035 (2)	0.0180 (18)	0.008 (2)	0.0186 (19)
C15A	0.079 (4)	0.039 (3)	0.066 (4)	0.007 (2)	0.023 (3)	0.028 (3)
C16A	0.092 (5)	0.041 (3)	0.066 (4)	0.000 (3)	0.029 (3)	0.018 (3)
O8A	0.0502 (18)	0.0399 (18)	0.058 (2)	0.0060 (14)	0.0111 (15)	0.0283 (17)
O9A	0.086 (3)	0.056 (2)	0.045 (3)	0.0240 (19)	−0.0081 (18)	0.029 (2)
C14C	0.062 (2)	0.041 (2)	0.035 (2)	0.0180 (18)	0.008 (2)	0.0186 (19)
C17A	0.031 (2)	0.045 (2)	0.037 (2)	−0.0028 (17)	0.0083 (18)	0.0103 (17)
C18A	0.0237 (19)	0.039 (2)	0.045 (2)	−0.0125 (16)	−0.0058 (17)	0.0109 (17)
C19A	0.048 (2)	0.040 (2)	0.045 (2)	0.0006 (18)	−0.0187 (19)	0.0182 (18)
C20A	0.040 (2)	0.031 (2)	0.053 (2)	0.0025 (17)	0.0037 (19)	0.0159 (18)
C21A	0.028 (2)	0.037 (2)	0.042 (2)	−0.0104 (16)	−0.0031 (18)	0.0116 (17)
O1A	0.0235 (13)	0.0267 (13)	0.0322 (13)	−0.0008 (10)	0.0002 (11)	0.0111 (10)
O2A	0.0262 (13)	0.0358 (14)	0.0401 (14)	−0.0113 (11)	−0.0106 (11)	0.0162 (11)
O3A	0.0353 (14)	0.0366 (14)	0.0402 (14)	−0.0039 (11)	−0.0151 (12)	0.0174 (11)
O4A	0.0312 (13)	0.0305 (13)	0.0328 (13)	0.0010 (11)	0.0014 (11)	0.0129 (11)
O5A	0.0242 (13)	0.0392 (14)	0.0430 (14)	−0.0094 (11)	−0.0072 (11)	0.0190 (12)
O6A	0.0270 (13)	0.0345 (14)	0.0308 (13)	−0.0001 (11)	−0.0059 (11)	0.0056 (11)
O7A	0.0474 (17)	0.0515 (17)	0.0362 (15)	0.0041 (13)	−0.0091 (13)	0.0152 (12)
C1B	0.031 (2)	0.0235 (18)	0.0268 (19)	0.0050 (15)	0.0044 (16)	0.0065 (15)
C2B	0.0217 (19)	0.0299 (19)	0.0254 (19)	0.0014 (15)	−0.0025 (15)	0.0064 (15)
C3B	0.027 (2)	0.0275 (19)	0.0281 (19)	−0.0012 (16)	0.0026 (16)	0.0098 (16)
C4B	0.0201 (19)	0.032 (2)	0.030 (2)	−0.0015 (15)	−0.0035 (16)	0.0046 (16)
C5B	0.025 (2)	0.033 (2)	0.0269 (19)	0.0029 (16)	−0.0060 (16)	0.0098 (16)
C6B	0.0258 (19)	0.0270 (19)	0.0212 (18)	0.0006 (15)	−0.0004 (15)	0.0053 (15)
C7B	0.0218 (19)	0.0243 (19)	0.0255 (19)	−0.0010 (15)	0.0015 (16)	0.0032 (15)
C8B	0.0250 (19)	0.030 (2)	0.0271 (19)	0.0028 (16)	−0.0025 (16)	0.0072 (16)
C9B	0.0215 (18)	0.0274 (19)	0.0268 (19)	−0.0036 (15)	−0.0034 (15)	0.0030 (15)
C10B	0.0207 (18)	0.032 (2)	0.0267 (19)	0.0052 (15)	−0.0044 (16)	0.0043 (15)
C11B	0.033 (2)	0.031 (2)	0.030 (2)	0.0091 (17)	−0.0041 (17)	0.0023 (16)
C12B	0.034 (2)	0.032 (2)	0.028 (2)	0.0053 (16)	−0.0066 (17)	0.0047 (16)
C13B	0.028 (2)	0.037 (2)	0.030 (2)	0.0044 (17)	0.0003 (17)	0.0112 (17)
C14B	0.030 (2)	0.042 (2)	0.030 (2)	0.0056 (18)	−0.0031 (18)	0.0050 (17)
C15B	0.052 (3)	0.042 (2)	0.050 (2)	0.0017 (19)	−0.004 (2)	0.024 (2)
C16B	0.046 (2)	0.045 (2)	0.060 (3)	−0.0077 (19)	−0.008 (2)	0.017 (2)
C17B	0.036 (2)	0.046 (2)	0.039 (2)	−0.0042 (17)	0.0026 (18)	0.0185 (18)
C18B	0.026 (2)	0.036 (2)	0.044 (2)	−0.0084 (16)	−0.0021 (18)	0.0121 (17)
C19B	0.029 (2)	0.049 (2)	0.047 (2)	−0.0016 (18)	−0.0154 (18)	0.0145 (19)
C20B	0.039 (2)	0.033 (2)	0.052 (2)	0.0032 (17)	0.0028 (19)	0.0205 (18)
C21B	0.0222 (19)	0.045 (2)	0.044 (2)	−0.0104 (17)	−0.0054 (17)	0.0095 (18)
O1B	0.0335 (14)	0.0268 (13)	0.0306 (13)	−0.0009 (11)	−0.0029 (11)	0.0090 (10)
O2B	0.0297 (14)	0.0345 (14)	0.0398 (14)	−0.0104 (11)	−0.0100 (11)	0.0155 (11)
O3B	0.0272 (13)	0.0440 (15)	0.0410 (15)	−0.0067 (11)	−0.0127 (11)	0.0188 (12)
O4B	0.0267 (13)	0.0292 (13)	0.0319 (13)	−0.0029 (10)	0.0003 (11)	0.0125 (11)
O5B	0.0281 (14)	0.0398 (14)	0.0428 (14)	−0.0120 (11)	−0.0098 (11)	0.0210 (12)
O6B	0.0365 (15)	0.0402 (15)	0.0485 (15)	−0.0080 (12)	−0.0234 (12)	0.0146 (12)
O7B	0.0422 (16)	0.0465 (17)	0.0394 (15)	−0.0008 (12)	−0.0118 (13)	0.0148 (12)
O8B	0.0370 (15)	0.0413 (15)	0.0463 (15)	0.0024 (12)	−0.0026 (12)	0.0242 (12)
O9B	0.0420 (16)	0.0589 (17)	0.0525 (17)	0.0091 (13)	−0.0066 (14)	0.0289 (14)

### Geometric parameters (Å, º)

**Table:**

C1A—C10A	1.373 (4)	C21A—H21B	0.9800
C1A—O1A	1.382 (3)	C21A—H21C	0.9800
C1A—C2A	1.436 (4)	O7A—H7A	0.8400
C2A—C3A	1.437 (4)	C1B—O1B	1.377 (3)
C2A—C7A	1.439 (4)	C1B—C10B	1.383 (4)
C3A—C4A	1.368 (4)	C1B—C2B	1.437 (4)
C3A—O2A	1.369 (3)	C2B—C3B	1.436 (4)
C4A—C5A	1.391 (4)	C2B—C7B	1.444 (4)
C4A—H4A	0.9500	C3B—C4B	1.369 (4)
C5A—O3A	1.364 (3)	C3B—O2B	1.370 (3)
C5A—C6A	1.381 (4)	C4B—C5B	1.393 (4)
C6A—O4A	1.376 (3)	C4B—H4B	0.9500
C6A—C7A	1.408 (4)	C5B—O3B	1.364 (3)
C7A—C8A	1.450 (4)	C5B—C6B	1.385 (4)
C8A—C9A	1.356 (4)	C6B—O4B	1.383 (3)
C8A—O5A	1.369 (3)	C6B—C7B	1.409 (4)
C9A—C10A	1.425 (4)	C7B—C8B	1.443 (4)
C9A—H9A	0.9500	C8B—C9B	1.350 (4)
C10A—C11A	1.479 (4)	C8B—O5B	1.373 (3)
C11A—O6A	1.268 (3)	C9B—C10B	1.417 (4)
C11A—C12A	1.446 (4)	C9B—H9B	0.9500
C12A—C13A	1.343 (4)	C10B—C11B	1.488 (4)
C12A—H12A	0.9500	C11B—O6B	1.260 (3)
C13A—O7A	1.321 (4)	C11B—C12B	1.443 (4)
C13A—C14A	1.518 (4)	C12B—C13B	1.362 (4)
C14A—O9A	1.200 (4)	C12B—H12B	0.9500
C14A—O8A	1.337 (4)	C13B—O7B	1.326 (4)
C15A—O8A	1.465 (4)	C13B—C14B	1.492 (4)
C15A—C16A	1.474 (5)	C14B—O9B	1.207 (4)
C15A—H15A	0.9900	C14B—O8B	1.337 (4)
C15A—H15B	0.9900	C15B—O8B	1.453 (3)
C16A—H16A	0.9800	C15B—C16B	1.500 (4)
C16A—H16B	0.9800	C15B—H15C	0.9900
C16A—H16C	0.9800	C15B—H15D	0.9900
C15C—O8C	1.470 (6)	C16B—H16D	0.9800
C15C—C16C	1.478 (7)	C16B—H16E	0.9800
C15C—H15E	0.9900	C16B—H16F	0.9800
C15C—H15F	0.9900	C17B—O1B	1.436 (3)
C16C—H16G	0.9800	C17B—H17D	0.9800
C16C—H16H	0.9800	C17B—H17E	0.9800
C16C—H16I	0.9800	C17B—H17F	0.9800
C17A—O1A	1.435 (3)	C18B—O2B	1.440 (3)
C17A—H17A	0.9800	C18B—H18D	0.9800
C17A—H17B	0.9800	C18B—H18E	0.9800
C17A—H17C	0.9800	C18B—H18F	0.9800
C18A—O2A	1.429 (3)	C19B—O3B	1.437 (3)
C18A—H18A	0.9800	C19B—H19D	0.9800
C18A—H18B	0.9800	C19B—H19E	0.9800
C18A—H18C	0.9800	C19B—H19F	0.9800
C19A—O3A	1.438 (3)	C20B—O4B	1.432 (3)
C19A—H19A	0.9800	C20B—H20D	0.9800
C19A—H19B	0.9800	C20B—H20E	0.9800
C19A—H19C	0.9800	C20B—H20F	0.9800
C20A—O4A	1.435 (3)	C21B—O5B	1.436 (3)
C20A—H20A	0.9800	C21B—H21D	0.9800
C20A—H20B	0.9800	C21B—H21E	0.9800
C20A—H20C	0.9800	C21B—H21F	0.9800
C21A—O5A	1.442 (3)	O7B—H7B	0.8400
C21A—H21A	0.9800		
			
C10A—C1A—O1A	119.0 (3)	C1A—O1A—C17A	113.2 (2)
C10A—C1A—C2A	121.5 (3)	C3A—O2A—C18A	118.5 (2)
O1A—C1A—C2A	119.4 (3)	C5A—O3A—C19A	118.3 (2)
C1A—C2A—C3A	123.2 (3)	C6A—O4A—C20A	112.5 (2)
C1A—C2A—C7A	119.7 (3)	C8A—O5A—C21A	117.1 (2)
C3A—C2A—C7A	117.0 (3)	C13A—O7A—H7A	109.5
C4A—C3A—O2A	121.4 (3)	O1B—C1B—C10B	118.9 (3)
C4A—C3A—C2A	121.8 (3)	O1B—C1B—C2B	119.7 (3)
O2A—C3A—C2A	116.9 (3)	C10B—C1B—C2B	121.2 (3)
C3A—C4A—C5A	119.8 (3)	C3B—C2B—C1B	123.8 (3)
C3A—C4A—H4A	120.1	C3B—C2B—C7B	117.0 (3)
C5A—C4A—H4A	120.1	C1B—C2B—C7B	119.2 (3)
O3A—C5A—C6A	116.0 (3)	C4B—C3B—O2B	121.0 (3)
O3A—C5A—C4A	122.5 (3)	C4B—C3B—C2B	121.5 (3)
C6A—C5A—C4A	121.5 (3)	O2B—C3B—C2B	117.6 (3)
O4A—C6A—C5A	117.1 (3)	C3B—C4B—C5B	120.5 (3)
O4A—C6A—C7A	122.8 (3)	C3B—C4B—H4B	119.7
C5A—C6A—C7A	120.1 (3)	C5B—C4B—H4B	119.7
C6A—C7A—C2A	119.8 (3)	O3B—C5B—C6B	115.5 (3)
C6A—C7A—C8A	123.7 (3)	O3B—C5B—C4B	123.7 (3)
C2A—C7A—C8A	116.6 (3)	C6B—C5B—C4B	120.8 (3)
C9A—C8A—O5A	122.6 (3)	O4B—C6B—C5B	117.4 (3)
C9A—C8A—C7A	121.5 (3)	O4B—C6B—C7B	122.3 (3)
O5A—C8A—C7A	115.9 (3)	C5B—C6B—C7B	120.3 (3)
C8A—C9A—C10A	122.1 (3)	C6B—C7B—C8B	123.0 (3)
C8A—C9A—H9A	119.0	C6B—C7B—C2B	119.8 (3)
C10A—C9A—H9A	119.0	C8B—C7B—C2B	117.1 (3)
C1A—C10A—C9A	118.5 (3)	C9B—C8B—O5B	122.3 (3)
C1A—C10A—C11A	125.1 (3)	C9B—C8B—C7B	121.5 (3)
C9A—C10A—C11A	116.3 (3)	O5B—C8B—C7B	116.2 (3)
O6A—C11A—C12A	118.2 (3)	C8B—C9B—C10B	122.2 (3)
O6A—C11A—C10A	117.4 (3)	C8B—C9B—H9B	118.9
C12A—C11A—C10A	124.4 (3)	C10B—C9B—H9B	118.9
C13A—C12A—C11A	120.3 (3)	C1B—C10B—C9B	118.8 (3)
C13A—C12A—H12A	119.8	C1B—C10B—C11B	126.6 (3)
C11A—C12A—H12A	119.8	C9B—C10B—C11B	114.6 (3)
O7A—C13A—C12A	124.9 (3)	O6B—C11B—C12B	117.6 (3)
O7A—C13A—C14A	112.6 (3)	O6B—C11B—C10B	117.2 (3)
C12A—C13A—C14A	122.5 (3)	C12B—C11B—C10B	125.2 (3)
O9A—C14A—O8A	127.0 (4)	C13B—C12B—C11B	119.9 (3)
O9A—C14A—C13A	122.6 (4)	C13B—C12B—H12B	120.1
O8A—C14A—C13A	110.4 (3)	C11B—C12B—H12B	120.1
O8A—C15A—C16A	110.4 (3)	O7B—C13B—C12B	125.3 (3)
O8A—C15A—H15A	109.6	O7B—C13B—C14B	112.0 (3)
C16A—C15A—H15A	109.6	C12B—C13B—C14B	122.8 (3)
O8A—C15A—H15B	109.6	O9B—C14B—O8B	124.5 (3)
C16A—C15A—H15B	109.6	O9B—C14B—C13B	123.8 (3)
H15A—C15A—H15B	108.1	O8B—C14B—C13B	111.7 (3)
C15A—C16A—H16A	109.5	O8B—C15B—C16B	107.3 (3)
C15A—C16A—H16B	109.5	O8B—C15B—H15C	110.3
H16A—C16A—H16B	109.5	C16B—C15B—H15C	110.3
C15A—C16A—H16C	109.5	O8B—C15B—H15D	110.3
H16A—C16A—H16C	109.5	C16B—C15B—H15D	110.3
H16B—C16A—H16C	109.5	H15C—C15B—H15D	108.5
C14A—O8A—C15A	116.9 (3)	C15B—C16B—H16D	109.5
O8C—C15C—C16C	108.3 (8)	C15B—C16B—H16E	109.5
O8C—C15C—H15E	110.0	H16D—C16B—H16E	109.5
C16C—C15C—H15E	110.0	C15B—C16B—H16F	109.5
O8C—C15C—H15F	110.0	H16D—C16B—H16F	109.5
C16C—C15C—H15F	110.0	H16E—C16B—H16F	109.5
H15E—C15C—H15F	108.4	O1B—C17B—H17D	109.5
C15C—C16C—H16G	109.5	O1B—C17B—H17E	109.5
C15C—C16C—H16H	109.5	H17D—C17B—H17E	109.5
H16G—C16C—H16H	109.5	O1B—C17B—H17F	109.5
C15C—C16C—H16I	109.5	H17D—C17B—H17F	109.5
H16G—C16C—H16I	109.5	H17E—C17B—H17F	109.5
H16H—C16C—H16I	109.5	O2B—C18B—H18D	109.5
O1A—C17A—H17A	109.5	O2B—C18B—H18E	109.5
O1A—C17A—H17B	109.5	H18D—C18B—H18E	109.5
H17A—C17A—H17B	109.5	O2B—C18B—H18F	109.5
O1A—C17A—H17C	109.5	H18D—C18B—H18F	109.5
H17A—C17A—H17C	109.5	H18E—C18B—H18F	109.5
H17B—C17A—H17C	109.5	O3B—C19B—H19D	109.5
O2A—C18A—H18A	109.5	O3B—C19B—H19E	109.5
O2A—C18A—H18B	109.5	H19D—C19B—H19E	109.5
H18A—C18A—H18B	109.5	O3B—C19B—H19F	109.5
O2A—C18A—H18C	109.5	H19D—C19B—H19F	109.5
H18A—C18A—H18C	109.5	H19E—C19B—H19F	109.5
H18B—C18A—H18C	109.5	O4B—C20B—H20D	109.5
O3A—C19A—H19A	109.5	O4B—C20B—H20E	109.5
O3A—C19A—H19B	109.5	H20D—C20B—H20E	109.5
H19A—C19A—H19B	109.5	O4B—C20B—H20F	109.5
O3A—C19A—H19C	109.5	H20D—C20B—H20F	109.5
H19A—C19A—H19C	109.5	H20E—C20B—H20F	109.5
H19B—C19A—H19C	109.5	O5B—C21B—H21D	109.5
O4A—C20A—H20A	109.5	O5B—C21B—H21E	109.5
O4A—C20A—H20B	109.5	H21D—C21B—H21E	109.5
H20A—C20A—H20B	109.5	O5B—C21B—H21F	109.5
O4A—C20A—H20C	109.5	H21D—C21B—H21F	109.5
H20A—C20A—H20C	109.5	H21E—C21B—H21F	109.5
H20B—C20A—H20C	109.5	C1B—O1B—C17B	114.9 (2)
O5A—C21A—H21A	109.5	C3B—O2B—C18B	117.6 (2)
O5A—C21A—H21B	109.5	C5B—O3B—C19B	118.0 (2)
H21A—C21A—H21B	109.5	C6B—O4B—C20B	113.9 (2)
O5A—C21A—H21C	109.5	C8B—O5B—C21B	116.8 (2)
H21A—C21A—H21C	109.5	C13B—O7B—H7B	109.5
H21B—C21A—H21C	109.5	C14B—O8B—C15B	115.7 (3)
			
C10A—C1A—C2A—C3A	179.7 (3)	O1B—C1B—C2B—C3B	6.8 (5)
O1A—C1A—C2A—C3A	1.4 (5)	C10B—C1B—C2B—C3B	−177.4 (3)
C10A—C1A—C2A—C7A	1.8 (4)	O1B—C1B—C2B—C7B	−174.0 (3)
O1A—C1A—C2A—C7A	−176.5 (3)	C10B—C1B—C2B—C7B	1.9 (4)
C1A—C2A—C3A—C4A	−175.9 (3)	C1B—C2B—C3B—C4B	179.5 (3)
C7A—C2A—C3A—C4A	2.1 (5)	C7B—C2B—C3B—C4B	0.2 (5)
C1A—C2A—C3A—O2A	5.2 (4)	C1B—C2B—C3B—O2B	−1.3 (5)
C7A—C2A—C3A—O2A	−176.8 (3)	C7B—C2B—C3B—O2B	179.4 (3)
O2A—C3A—C4A—C5A	177.6 (3)	O2B—C3B—C4B—C5B	−177.8 (3)
C2A—C3A—C4A—C5A	−1.2 (5)	C2B—C3B—C4B—C5B	1.4 (5)
C3A—C4A—C5A—O3A	−179.3 (3)	C3B—C4B—C5B—O3B	178.5 (3)
C3A—C4A—C5A—C6A	−0.5 (5)	C3B—C4B—C5B—C6B	−0.3 (5)
O3A—C5A—C6A—O4A	1.1 (4)	O3B—C5B—C6B—O4B	−4.9 (4)
C4A—C5A—C6A—O4A	−177.8 (3)	C4B—C5B—C6B—O4B	173.9 (3)
O3A—C5A—C6A—C7A	−179.9 (3)	O3B—C5B—C6B—C7B	178.7 (3)
C4A—C5A—C6A—C7A	1.3 (5)	C4B—C5B—C6B—C7B	−2.5 (5)
O4A—C6A—C7A—C2A	178.7 (3)	O4B—C6B—C7B—C8B	7.8 (5)
C5A—C6A—C7A—C2A	−0.3 (5)	C5B—C6B—C7B—C8B	−176.0 (3)
O4A—C6A—C7A—C8A	−1.5 (5)	O4B—C6B—C7B—C2B	−172.2 (3)
C5A—C6A—C7A—C8A	179.5 (3)	C5B—C6B—C7B—C2B	4.0 (4)
C1A—C2A—C7A—C6A	176.7 (3)	C3B—C2B—C7B—C6B	−2.9 (4)
C3A—C2A—C7A—C6A	−1.3 (4)	C1B—C2B—C7B—C6B	177.8 (3)
C1A—C2A—C7A—C8A	−3.1 (4)	C3B—C2B—C7B—C8B	177.2 (3)
C3A—C2A—C7A—C8A	178.9 (3)	C1B—C2B—C7B—C8B	−2.1 (4)
C6A—C7A—C8A—C9A	−177.7 (3)	C6B—C7B—C8B—C9B	−178.8 (3)
C2A—C7A—C8A—C9A	2.1 (4)	C2B—C7B—C8B—C9B	1.1 (4)
C6A—C7A—C8A—O5A	1.5 (4)	C6B—C7B—C8B—O5B	2.8 (4)
C2A—C7A—C8A—O5A	−178.7 (3)	C2B—C7B—C8B—O5B	−177.2 (3)
O5A—C8A—C9A—C10A	−178.9 (3)	O5B—C8B—C9B—C10B	178.5 (3)
C7A—C8A—C9A—C10A	0.2 (5)	C7B—C8B—C9B—C10B	0.3 (5)
O1A—C1A—C10A—C9A	178.9 (3)	O1B—C1B—C10B—C9B	175.4 (3)
C2A—C1A—C10A—C9A	0.6 (4)	C2B—C1B—C10B—C9B	−0.5 (5)
O1A—C1A—C10A—C11A	3.6 (5)	O1B—C1B—C10B—C11B	−7.6 (5)
C2A—C1A—C10A—C11A	−174.8 (3)	C2B—C1B—C10B—C11B	176.5 (3)
C8A—C9A—C10A—C1A	−1.6 (5)	C8B—C9B—C10B—C1B	−0.6 (5)
C8A—C9A—C10A—C11A	174.2 (3)	C8B—C9B—C10B—C11B	−178.0 (3)
C1A—C10A—C11A—O6A	162.6 (3)	C1B—C10B—C11B—O6B	−171.3 (3)
C9A—C10A—C11A—O6A	−12.9 (4)	C9B—C10B—C11B—O6B	5.8 (4)
C1A—C10A—C11A—C12A	−19.7 (5)	C1B—C10B—C11B—C12B	8.8 (5)
C9A—C10A—C11A—C12A	164.8 (3)	C9B—C10B—C11B—C12B	−174.0 (3)
O6A—C11A—C12A—C13A	0.7 (5)	O6B—C11B—C12B—C13B	5.3 (5)
C10A—C11A—C12A—C13A	−177.0 (3)	C10B—C11B—C12B—C13B	−174.9 (3)
C11A—C12A—C13A—O7A	0.0 (5)	C11B—C12B—C13B—O7B	−3.2 (5)
C11A—C12A—C13A—C14A	178.8 (3)	C11B—C12B—C13B—C14B	178.6 (3)
O7A—C13A—C14A—O9A	19.6 (6)	O7B—C13B—C14B—O9B	−20.1 (5)
C12A—C13A—C14A—O9A	−159.3 (4)	C12B—C13B—C14B—O9B	158.3 (3)
O7A—C13A—C14A—O8A	−160.7 (3)	O7B—C13B—C14B—O8B	159.6 (3)
C12A—C13A—C14A—O8A	20.4 (5)	C12B—C13B—C14B—O8B	−21.9 (4)
O9A—C14A—O8A—C15A	−0.3 (7)	C10B—C1B—O1B—C17B	92.4 (3)
C13A—C14A—O8A—C15A	−180.0 (3)	C2B—C1B—O1B—C17B	−91.6 (3)
C16A—C15A—O8A—C14A	−95.6 (5)	C4B—C3B—O2B—C18B	−3.9 (4)
C10A—C1A—O1A—C17A	−94.7 (3)	C2B—C3B—O2B—C18B	176.9 (3)
C2A—C1A—O1A—C17A	83.7 (3)	C6B—C5B—O3B—C19B	178.7 (3)
C4A—C3A—O2A—C18A	6.0 (4)	C4B—C5B—O3B—C19B	−0.1 (5)
C2A—C3A—O2A—C18A	−175.1 (3)	C5B—C6B—O4B—C20B	82.7 (3)
C6A—C5A—O3A—C19A	180.0 (3)	C7B—C6B—O4B—C20B	−101.0 (3)
C4A—C5A—O3A—C19A	−1.2 (4)	C9B—C8B—O5B—C21B	2.1 (4)
C5A—C6A—O4A—C20A	−87.5 (3)	C7B—C8B—O5B—C21B	−179.6 (3)
C7A—C6A—O4A—C20A	93.5 (3)	O9B—C14B—O8B—C15B	4.2 (5)
C9A—C8A—O5A—C21A	2.6 (4)	C13B—C14B—O8B—C15B	−175.5 (3)
C7A—C8A—O5A—C21A	−176.6 (3)	C16B—C15B—O8B—C14B	178.6 (3)

### Hydrogen-bond geometry (Å, º)

Cg1, Cg2 and Cg3 are the centroids of the C1*A*/C2*A*/C7*A*–C10*A*, C2*A*–C7*A* and C2*B*–C7*B* rings, respectively.

**Table:**

*D*—H···*A*	*D*—H	H···*A*	*D*···*A*	*D*—H···*A*
O7*A*—H7*A*···O6*A*	0.84	1.77	2.499 (3)	144
O7*B*—H7*B*···O6*B*	0.84	1.77	2.497 (3)	143
C17*B*—H17*D*···O6*B*^i^	0.98	2.43	3.234 (4)	139
C19*A*—H19*B*···O9*B*^ii^	0.98	2.45	3.419 (4)	168
C19*B*—H19*E*···O9*A*^iii^	0.98	2.47	3.440 (5)	172
C20*A*—H20*B*···O4*B*^iv^	0.98	2.50	3.384 (4)	150
C20*B*—H20*E*···O4*A*^v^	0.98	2.57	3.382 (4)	140
C20*B*—H20*F*···O9*B*^i^	0.98	2.58	3.460 (4)	150
C18*A*—H18*A*···*Cg*3^vi^	0.98	2.66	3.486 (3)	142
C18*A*—H18*C*···*Cg*1^vi^	0.98	2.92	3.844 (3)	158
C18*B*—H18*F*···*Cg*2^vii^	0.98	2.66	3.522 (3)	147
C21*A*—H21*A*···*Cg*2^vii^	0.98	2.96	3.845 (3)	150

Symmetry codes: (i) −*x*+1, −*y*+2, −*z*+1; (ii) −*x*+2, −*y*+1, −*z*+1; (iii) −*x*, −*y*+2, −*z*; (iv) *x*, *y*−1, *z*; (v) *x*, *y*+1, *z*; (vi) *x*+1, *y*, *z*; (vii) *x*−1, *y*, *z*.
